# The Graft Infusion Technique (GIT) for Treatment of Peri-Implantitis Defects: Case Series

**DOI:** 10.30476/DENTJODS.2021.86658.1203

**Published:** 2021-12

**Authors:** Neel B. Bhatavadekar, Amit S. Gharpure

**Affiliations:** 1 Private Practice, Clarus Dental Specialities, Pune, India. Adjunct Faculty, University of North Carolina at Chapel Hill, NC, Adjunct Faculty, University of Texas Health Science Center, Houston, TX, Adjunct Faculty, Bioengineering Department, Rice University, FL; 2 Graduate Periodontics, University of Washington School of Dentistry, Seattle, WA

**Keywords:** Dental Implants, Bone Grafting, Peri-implantitis, Radiographs, Surgical Procedures, Reconstructive Surgical Procedures, Deproteinized bovine bone mineral with 10% collagen, Success, Survival

## Abstract

Peri-implantitis is a site-specific infectious disease that causes an inflammatory process in soft tissues, and bone loss around an osseointegrated implant in function. Several techniques with non‐surgical or surgical debridement and decontamination followed by ongoing supportive therapy or regeneration of the peri‐implant bone defects have been proposed in the literature. However, the literature is still unclear on an effective protocol for implant surface decontamination or the appropriate choice of regenerative materials. This case series describes a surgical technique to treat peri-implantitis osseous defects using a mixture of deproteinized bovine bone mineral with 10% porcine collagen (DBBM-C) in a block form, soaked in an appropriate antibiotic. The use of this combination provides advantages such as good graft adaptability along with localized antibiotic release without the use of systemic antibiotics. Thus, this technique might be an effective method to treat amenable peri-implantitis defects. Additionally, the proposed algorithm also allows for customized culture based antibiotic loading. To the best of the authors’ knowledge, this is the first case series documenting this technique for peri-implantitis defects. Long-term studies with controlled samples would be necessary for further evaluation.

## Introduction

Peri‐implantitis is an inflammatory process affecting the surrounding peri‐implant tissues, which can result in loss of supporting bone structure [ 1 ]. The diagnostic criteria determines the prevalence of peri-implantitis, and has been shown to range from 6.6% [ 2 ], to 36.6% after an average of 8 years of implant loading [ 3 - 5 ]. With an increase in the number of implants being placed every year, there is a pressing need to develop an effective and predictable treatment for peri‐implantitis. 

The pathophysiology of peri-implantitis has been shown to have a more accelerated rate of progression compared to periodontitis [ 6 ], which is why a more aggressive treatment approach is often warranted [ 7 ]. The Sixth European Workshop on Periodontology concluded that non‐surgical therapy alone appears to be insufficient in the treatment of most cases of peri‐implantitis and surgical therapy is usually necessary [ 7 ]. 

Various treatments involving non‐surgical or surgical debridement and decontamination followed by ongoing supportive therapy [ 8 - 9 ], or regeneration of the peri‐implant bone defect [ 9 - 10 ], application of antibiotics such as tetracycline to the implant surface [ 11 - 12 ], have been described in the available literature. However, the most effective protocol for implant surface decontamination and appropriate choice of regenerative materials is still unclear in the literature [ 13 ]. There have been several studies on surgical therapies, which have looked at the use of bone grafts / bone substitutes with and without membranes, and they have documented clinical and radiographic improvements for at least 3 years from the time of treatment [ 14 - 17 ]. The grafting materials, timing and surgical technique varied in the studies and it was difficult to determine the superior method [ 10 ]. Unfortunately, none of the available techniques have been established as being predictable in achieving successful clinical and radiograph outcomes in the long-term, and further research in relation to the regenerative treatment of peri‐implantitis is warranted [ 10 , 13 ]. 

A mixture of deproteinized bovine bone mineral with porcine collagen in a block form (DBBM-C, Geistlich Bio-Oss^®^ Collagen, Geistlich Pharma AG, Wolhusen, Switzerland) has been successfully used in the literature for ridge preservation and guided tissue regeneration with stable results [ 18 - 19 ]. However, there is only one reported study, which evaluated the use of this material for grafting peri-implantitis defects [ 20 ]. The present clinical case series seeks to evaluate the 5-6 month clinical and radiographic results of DBBM-C soaked in an antibiotic covered with a native bilayer resorbable collagen membrane (Geistlich Bio-Gide^®^, Geistlich Pharma AG, Wolhusen, Switzerland) for surgical regenerative therapy of peri‐implantitis, using a single procedure approach.

## Case Presentation 1

A 47-year-old non-smoker female patient with no significant medical history, presented to the dental office for evaluation of bone loss around implant #18. Tooth #18 was extracted at the referring dental office 5 years prior, due to endodontic failure and was grafted with freeze-dried bone allograft and collagen membrane. 5 months later, after confirming adequate radiographic bone fill, a 4.6x10.5mm external implant (BioHorizons, Birmingham, AL, USA) was placed as per manufacturer’s recommendations using a 1-stage procedure and loaded with a definitive screw-retained restoration after 3 months. The patient was kept on a 6-month recall at the referring dental office. 5 years later, the patient was referred for evaluation of bone loss around #18 implant.

**Figure 1:**
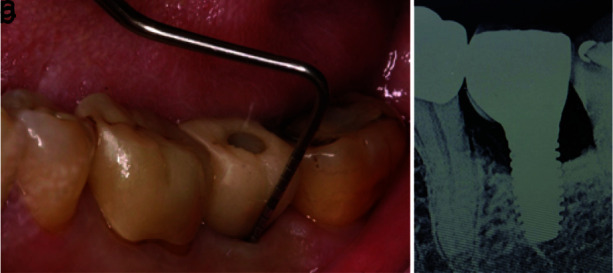
**a:** Buccal view of #17-20 showing 6mm probing depth on the facial surface of #18 implant. **b:** Initial radiograph with a crater-like defect and 40% bone loss around #18

At the initial consultation appointment, a detailed periodontal exam was conducted. Her periodontal findings were normal with only isolated areas of mild gingival inflammation; however, #18 implant had deep probing depths of 6mm on the facial and lingual with spontaneous bleeding on probing but no suppuration (Figure 1a). Medical history was non-contributory. Radiographic examination revealed a circumferential crater defect with bone loss up to 40% of the implant length (Figure 1b, 8). The patient was diagnosed with moderate peri-implantitis as per the Froum and Rosen [ 21 ] classification.

**Figure 2:**
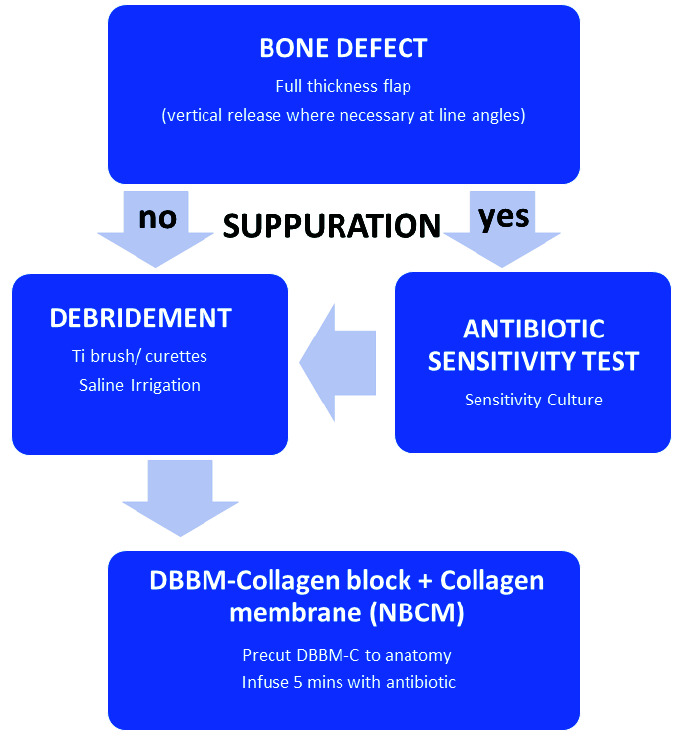
Flow-chart showing treatment protocol for treating peri-implant defect

Following the initial consultation, a treatment plan involving non-surgical initial therapy, surgical intervention and three-month maintenance protocol was discussed and the patient consented for treatment. Initial therapy involved scaling and root planning using ultrasonic and hand scalers for the natural dentition and use of titanium hand curettes for implant #18 followed by oral hygiene instructions. A 6-week re-evaluation revealed no change in probing depths and bleeding on probing for site #18, but there was decrease in plaque scores and gingival indices in the rest of the natural dentition. At this point, it was decided to treat site #18 surgically. A flow chart of treatment protocol is depicted in Figure 2. 

**Figure 3:**
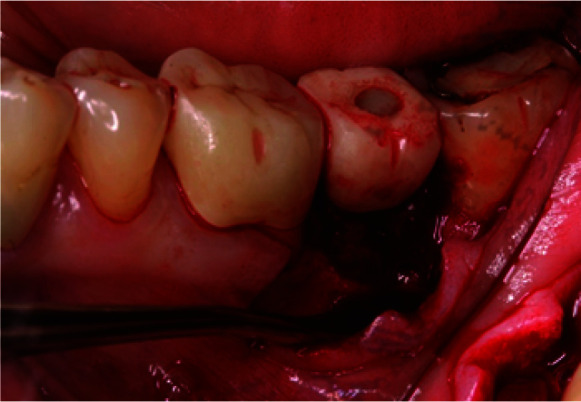
Sulcular incision from #17-18 with a vertical release on the distal line angle of #19 and reflection of full thickness flap

**Figure 4:**
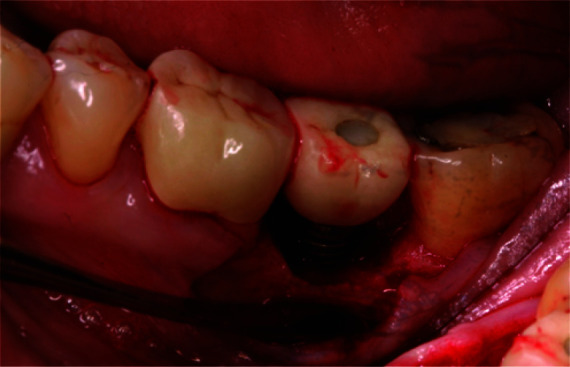
Full thickness flap elevation and removal of granulomatous tissue to visualize circumferential crater like defect around #19

**Figure 5:**
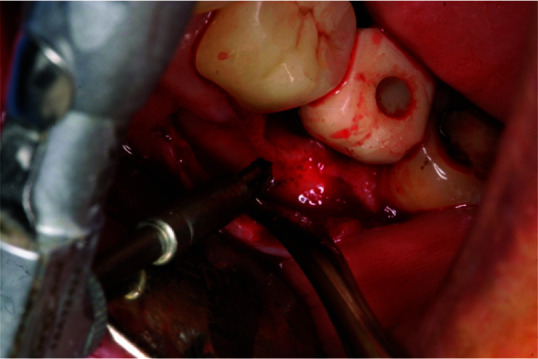
Use of titanium brush and saline for surface debridement

After infiltration with 2 carpules of 2% Lidocaine with 1:100,000 epinephrine, a sulcular incision was made on the facial and lingual surfaces of #17 and #18 and the lingual of #19, with a vertical release beyond the muco-gingival junction on the distofacial line angle of #19 (Figure 3). A full thickness flap was elevated, and titanium curettes were used to instrument the implant surface to remove granulomatous tissue (Figure 4). A titanium brush using a slow speed handpiece was used for cleaning the implant surface using adequate saline irrigation and a high vacuum suction (Figure 5).

**Figure 6:**
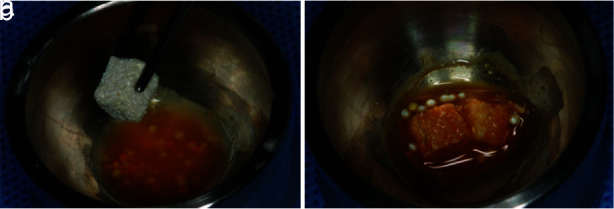
**a:** A mixture of 90% deproteinized bovine bone mineral with 10% porcine collagen in a block form of dimensions 10×10×8mm, **b:** Graft soaked in tetracycline and saline solution for 30 minutes

**Figure 7:**
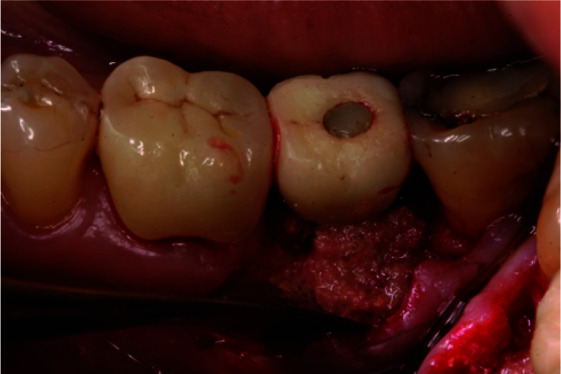
Graft placed in the defect and adapted to the dimensions of the defect. Subsequently, a resorbable membrane (NCBM) was placed over the graft (not shown in this image)

**Figure 8:**
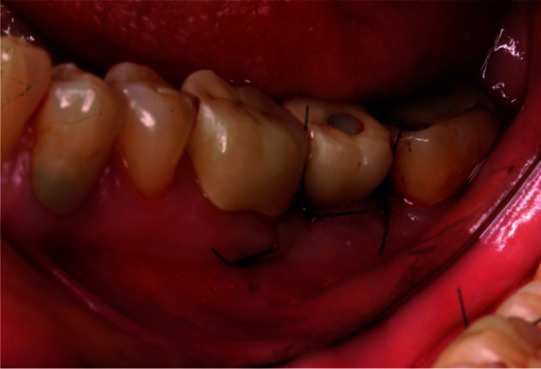
Flap sutured with 5-0 polyamide to obtain primary closure and prevent exposure of graft and membrane

The implant surface was then rinsed thoroughly with normal saline and a circumferential crater like bony defect was visualized. A mixture of DBBM-C in a block form of dimensions 10×10×8mm was soaked for 30 minutes using tetracycline 400mg in 1ml saline (Figure 6) as reported in the literature [ 11 - 12 ]. The block was then shaped to conform to the defect shape, and the site was grafted with the block to fill the defect completely and the graft was covered with a native bilayer resorbable collagen membrane (Figure 7). The flap was closed such that it completely covered the graft and the membrane; it was sutured in place using 5-0 polyamide single interrupted sutures (Figure 8). 

**Figure 9:**
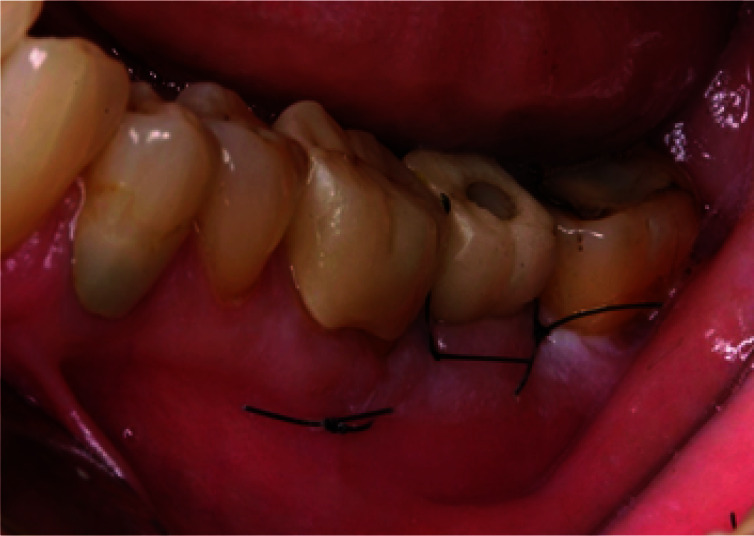
3-week follow-up showing intact sutures, minimal inflammation and no exposure of graft material

**Figure 10:**
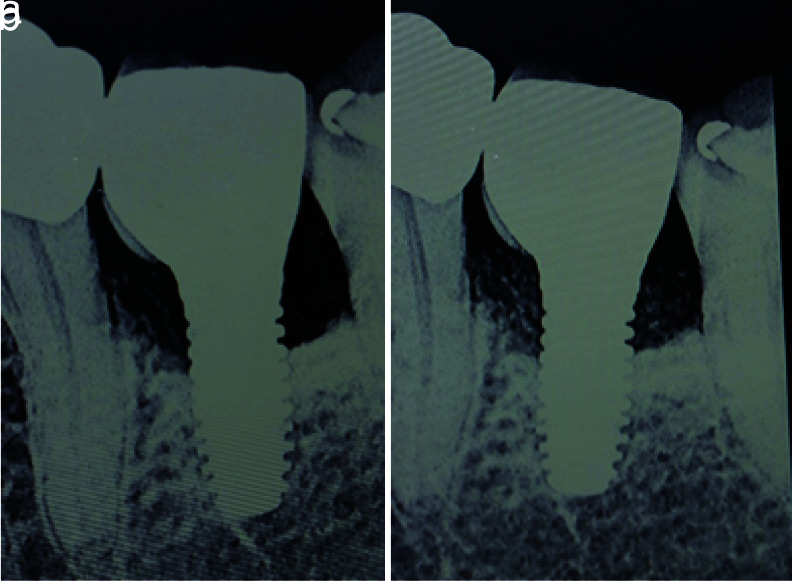
**a:** Pre-operative radiograph and **b** post-operative radiograph (taken at 5 months)

Pain control included using Ibuprofen 600 mg every 6–8 hours for days 1 and 2 and then as needed after that. After the surgery, the patient was asked not to brush the surgical site for 7 days and use a soft brushing gently for the following 14 days. For the first week, the patient was prescribed 0.12% chlorhexidine mouthwash, which was to be used twice daily. The patient was seen at 1 week, 2 weeks, 3 weeks (Figure 9) and 5 months after the procedure. Healing appeared to proceed uneventfully, and sutures were removed at 3 weeks. A periapical radiograph was taken at 5 months and it showed complete bone-fill in the grafted defect (Figure 10). She is currently on a 4-month perio maintenance schedule. Probing depths have been reduced to 4mm on both the facial and the lingual and bleeding on probing is absent.

## Case Presentation 2

A 66-year-old healthy, non-smoker female patient with no significant medical history, presented to the dental office for evaluation of bone loss and suppuration around implant #30. Tooth #30 was extracted at the referring dental office 6 years prior, due to a vertical root fracture and was grafted with freeze-dried bone allograft and a collagen plug. 4.5 months later, after confirming adequate radiographic bone fill, a 4.5x11mm implant (Ankylos, Friadent, GmbH, Mannheim, Germany) was placed as per manufacturer’s recommendations using a 1-stage procedure and loaded with a definitive screw-retained restoration after 3 months. The patient was kept on a 6-month recall at the referring dental office. 4 years later, the patient was referred for evaluation of bone loss and suppuration around #30 implant.

**Figure 11:**
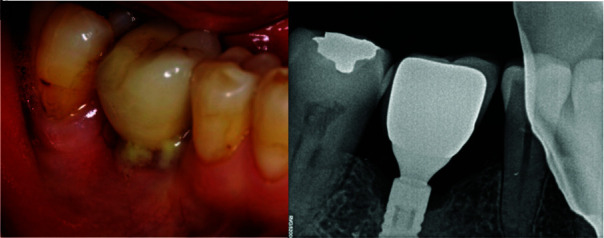
**a:** Buccal view of #30 with a 7mm probing depth and suppuration on the facial surface, **b:** Initial radiograph with a crater-like defect and 40% bone loss around #30

Her initial appointment was for a consultation and periodontal exam. Her periodontal findings were normal; however, #30 implant had deep probing depths of 7mm on the facial and lingual with spontaneous bleeding on probing and suppuration (Figure 11a). Radiographic examination revealed a circumferential crater defect with bone loss up to 40% of the implant length (Figure 11b). The patient was diagnosed with moderate peri-implantitis as per the Froum and Rosen [ 21 ] classification.

**Figure 12:**
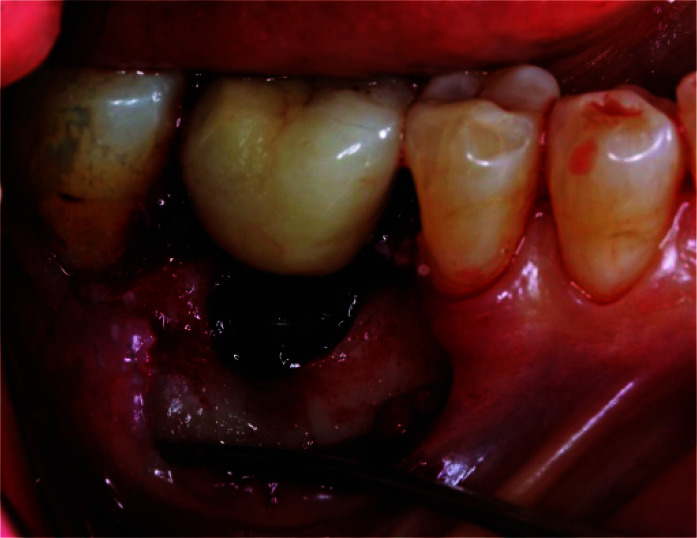
Sulcular incision from #30-31 with a vertical release on the mesial of #30 and reflection of full thickness flap to visualize the circumferential crater-like defect

Following the initial consultation, a treatment plan involving non-surgical initial therapy, surgical intervention and three-month maintenance protocol was discussed and the patient consented for treatment. Samples were obtained from the site showing suppuration with paper points for antibiotic sensitivity culture. Initial therapy involved scaling and root planning using ultrasonic and hand scalers for the natural dentition and use of titanium hand curettes for implant #30 followed by oral hygiene instructions. A 6-week re-evaluation revealed no change in probing depths and bleeding on probing for site #30. At this point, it was decided to treat site #30 using surgical intervention. A flow chart of treatment protocol is depicted in Figure 2. After infiltration with 2 carpules of 2% Lidocaine with 1:100,000 epinephrine, a sulcular incision was made on the facial and lingual surfaces of #29 and #31 with a vertical release beyond the muco-gingival junction on the mesial surface of #30 (Figure 12).

**Figure 13:**
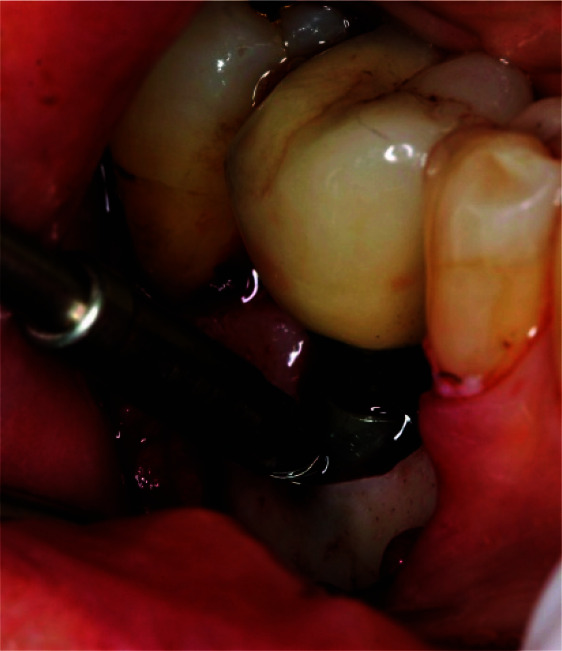
Use of titanium brush and saline for surface implantoplasty to obtain smooth surface

A full thickness flap was elevated, and titanium curettes were used to instrument the implant surface to remove granulomatous tissue. A titanium brush using a slow speed handpiece was used for cleaning the implant surface using adequate saline irrigation and a high vacuum suction (Figure 13). The implant surface was rinsed thoroughly with normal saline and a circumferential crater like bony defect was visualized. The results of antibiotic sensitivity culture test demonstrated the susceptibility of microbial flora to Amoxicillin. 

**Figure 14:**
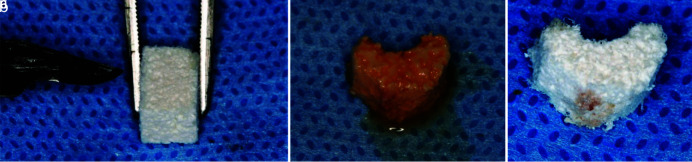
**a:** A mixture of 90% deproteinized bovine bone mineral with 10% porcine collagen (DBBM-C) in a block form of dimensions 10×10×8mm, **b:** Graft pre-contoured, and **c:** soaked in amoxicillin and saline solution for 30 minutes

Based on these results, DBBM- C in a block form of dimensions 10×10×8mm was pre-contoured to the fit the defect and soaked for 30 minutes in a solution of 500 mg Amoxycillin with 1ml saline) (Figure 14a,b,c). 

**Figure 15:**
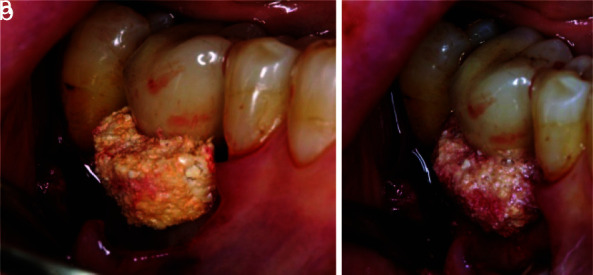
**a:** Graft placed in the defect and **b:** adapted to the dimensions of the defect and infused in the antibiotic solution

**Figure 16:**
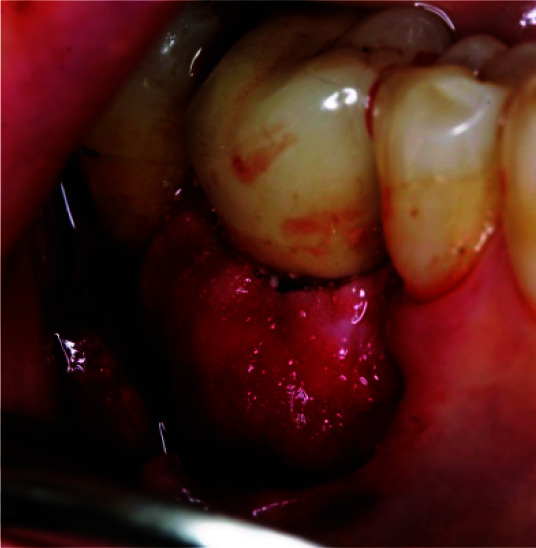
A porcine resorbable collagen membrane (NBCM) was placed over the graft

**Figure 17:**
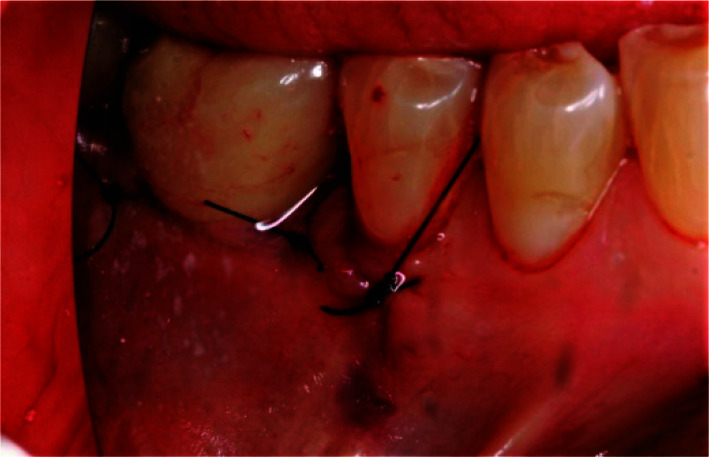
Flap sutured with 5-0 polyamide to obtain primary closure and prevent exposure of graft and membrane

**Figure 18:**
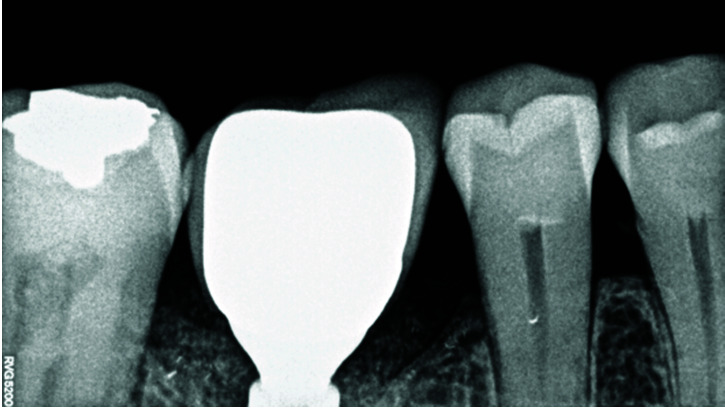
Immediate post-op radiograph demonstrating the grafted material in the defect

The site was grafted such that the block completely filled the defect (Figure 15a,b) and was covered with a native bilayer resorbable collagen membrane (Figure 16). The flap was closed such that it completely covered the graft and the membrane and sutured in place using 5-0 polyamide single interrupted sutures (Figure 17) and an immediate post-operative radiograph was taken to visualize the bone graft (Figure 18). Pain control included using Ibuprofen 600 mg every 6–8 hours for days 1 and 2 and then as needed after that. After the surgery, the patient was asked not to brush the surgical site for 7 days and use a soft brushing gently for the folowing14 days. For the first week, patient was prescribed 0.12% chlorhexidine mouthwash, which was to be used twice daily. 

**Figure 19:**
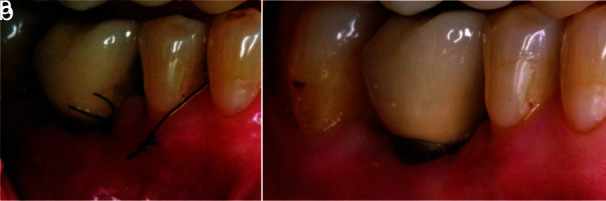
**a:** 2 weeks of follow-op showing intact sutures, minimal inflammation and no exposure of graft material, **b:** 2-month follow-up demonstrating mild recession. Probing depth 3mm, no BOP

**Figure 20:**
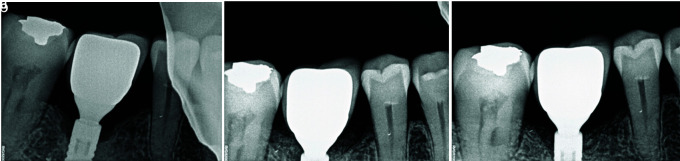
**a:** Pre-operative, **b:** immediate post-operative radiograph, **c:** radiograph at 6 months

Patient was seen at 1 week, 2 weeks (Figure 19a), 4 weeks (Figure 19b) and 6 months after the procedure. Healing appeared to proceed uneventfully, and sutures were removed at 3 weeks. A periapical radiograph was taken at 6 months and it showed bone-fill in the grafted defect (Figure 20a,b,c), with a clinical reduction of 3mm of probing depth. She is currently on a 4-month periodontal maintenance schedule. Probing depths have been reduced to 4mm on both the facial and the lingual and bleeding on probing is absent.

## Discussion

The biggest challenge in the treatment of peri-implantitis is surface decontamination. Roughened surfaces pose difficulty in thorough mechanical debridement. Additionally, applying an antibiotic solution directly to the implant surface after debridement has been shown to leave drug residues which might interfere with re-osseointegration and act as a reservoir of microorganisms [ 22 ]. In the GIT technique, the DBBM-C is soaked in an appropriate antibiotic (either sensitivity-specific antibiotic based on culture, or tetracycline [ 11 - 12 ], where culture is not possible). This may assist in providing localized antibiotic delivery, while using an osteoconductive carrier for future regeneration. A prior study evaluated the use of deproteinized bovine bone mineral in combination with tetracycline with beneficial effects [ 23 ]. However, this study also used systemic antibiotics, whereas the two cases in our study have been performed without systemic antibiotic loading. In cases with suppuration, the use of antibiotic sensitivity culture may provide an added benefit in selection of appropriate antibiotic during initial and surgical therapy, since prior reports have recommended specific targeted drug delivery for peri-implantitis when possible [ 24 ]. It would also be interesting to evaluate if DBBM-C soaked in an antibiotic solution provides a slightly more sustained release over a period as opposed to using particulate grafts with antibiotics. This aspect, however, was not evaluated in this case series, and it can be a subject for future research. In effect, with the GIT technique, a single stage treatment of patient can be performed where debridement, cleaning the implant surface, and subsequent regenerative procedure with graft soaked with appropriate antibiotic and membrane can be carried out together. However, like any preliminary case series, this study does have certain limitations.

The results cannot be extrapolated to serve as a general guideline for peri-implantitis. Compared to prior studies using particulate grafts, this technique is different in terms of using a shaped DBBM-C to fit around the defect snugly. Peri-implantitis defects have complex morphology, frequently with an intra-osseous component [ 25 ]. The graft used in this case series is easy to adapt and retain around such complex defects and is best suited for well-contained angular defects. This graft also provides good stability and volume maintenance, which are critical for regeneration. With advantages such as localized antibiotic release and good adaptability, the use of DBBM-C soaked in antibiotic may be an effective technique to treat peri-implant defects, using a single procedure approach. Long-term studies with controlled samples would be necessary for further evaluation of this technique. This case report was written after obtaining informed consent from the patient.

## Acknowledgement

The authors report no conflicts of interest related to the study.

## Conflict of Interest

The authors report no conflicts of interest related to this work 

## References

[1] Zitzmann NU, Berglundh T ( 2008). Definition and prevalence of peri-implant diseases. J Clin Periodontol.

[2] Mombelli A, Muller N, Cionca N ( 2012). The epidemiology of peri-implantitis. Clin Oral Implants Res.

[3] Atieh MA, Alsabeeha NH, Faggion CM  Jr, Duncan WJ ( 2013). The frequency of peri-implant diseases: a systematic review and meta-analysis. J Periodontol.

[4] Fagan MC, Owens H, Smaha J, Kao RT ( 2008). Simultaneous hard and soft tissue augmentation for implants in the esthetic zone: report of 37 consecutive cases. J Periodontol.

[5] Koldsland OC, Scheie AA, Aass AM ( 2010). Prevalence of peri-implantitis related to severity of the disease with different degrees of bone loss. J Periodontol.

[6] Carcuac O, Berglundh T ( 2014). Composition of human peri-implantitis and periodontitis lesions. J Dent Res.

[7] Lindhe J, Meyle J ( 2008). Peri-implant diseases: Consensus Report of the Sixth European Workshop on Periodontology. J Clin Periodontol.

[8] Heitz-Mayfield LJA, Salvi GE, Mombelli A, Loup PJ, Heitz F, Kruger E, et al ( 2018). Supportive peri-implant therapy following anti-infective surgical peri-implantitis treatment: 5-year survival and success. Clin Oral Implants Res.

[9] Heitz-Mayfield LJA, Salvi GE, Mombelli A, Faddy M, Lang NP ( 2012). Anti-infective surgical therapy of peri-implantitis. A 12-month prospective clinical study. Clin Oral Implants Res.

[10] Esposito M, Grusovin MG, Worthington HV ( 2012). Interventions for replacing missing teeth: treatment of peri-implantitis. The Cochrane database of systematic reviews. Cochrane Database Syst Rev.

[11] La Monaca G, Pranno N, Annibali S, Cristalli MP, Polimeni A ( 2018). Clinical and radiographic outcomes of a surgical reconstructive approach in the treatment of peri-implantitis lesions: A 5-year prospective case series. Clin Oral Implants Res.

[12] Mensi M, Scotti E, Calza S, Pilloni A, Grusovin MG, Mongardini C ( 2017). A new multiple anti-infective non-surgical therapy in the treatment of peri-implantitis: a case series. Minerva Stomatol.

[13] Tomasi C, Regidor E, Ortiz-Vigon A, Derks J ( 2019). Efficacy of reconstructive surgical therapy at peri-implantitis-related bone defects. A systematic review and meta-analysis. J Clin Periodontol.

[14] Behneke A, Behneke N, d'Hoedt B ( 2000). Treatment of peri-implantitis defects with autogenous bone grafts: six-month to 3-year results of a prospective study in 17 patients. Int J Oral Maxillofac Implants.

[15] Froum SJ, Froum SH, Rosen PS ( 2015). A Regenerative Approach to the Successful Treatment of Peri-implantitis: A Consecutive Series of 170 Implants in 100 Patients with 2- to 10-Year Follow-up. Int J Periodontics Restorative Dent.

[16] Roos-Jansaker AM, Lindahl C, Persson GR, Renvert S ( 2011). Long-term stability of surgical bone regenerative procedures of peri-implantitis lesions in a prospective case-control study over 3 years. J Clin Periodontol.

[17] Schwarz F, John G, Schmucker A, Sahm N, Becker J ( 2017). Combined surgical therapy of advanced peri-implantitis evaluating two methods of surface decontamination: a 7-year follow-up observation. J Clin Periodontol.

[18] Nevins ML, Camelo M, Lynch SE, Schenk RK, Nevins M ( 2003). Evaluation of periodontal regeneration following grafting intrabony defects with bio-oss collagen: a human histologic report. Int J Periodontics Restorative Dent.

[19] Cardaropoli D, Tamagnone L, Roffredo A, Gaveglio L, Cardaropoli G ( 2012). Socket preservation using bovine bone mineral and collagen membrane: a randomized controlled clinical trial with histologic analysis. Int J Periodontics Restorative Dent.

[20] Mercado F, Hamlet S, Ivanovski S ( 2018). Regenerative surgical therapy for peri-implantitis using deproteinized bovine bone mineral with 10% collagen, enamel matrix derivative and Doxycycline-A prospective 3-year cohort study. Clin Oral Implants Res.

[21] Froum SJ, Rosen PS ( 2012). A proposed classification for peri-implantitis. Int J Periodontics Restorative Dent.

[22] Lee JB, Kweon HH, Cho HJ, Kim CS, Kim YT ( 2019). Characteristics of Local Delivery Agents for Treating Peri-Implantitis on Dental Implant Surfaces: A Preclinical Study. J Oral Implantol.

[23] Dashti A, Ready D, Salih V, Knowles JC, Barralet JE, Wilson M, et al ( 2010). In vitro antibacterial efficacy of tetracycline hydrochloride adsorbed onto Bio-Oss bone graft. Journal of Biomedical Materials Research Part B, Applied Biomaterials.

[24] Rams TE, Degener JE, van Winkelhoff  AJ ( 2014). Antibiotic resistance in human peri-implantitis microbiota. Clin Oral Implants Res.

[25] Monje A, Pons R, Insua A, Nart J, Wang HL, Schwarz F ( 2019). Morphology and severity of peri-implantitis bone defects. Clin Implant Dent Relat Res.

